# Supplementary material for: Are carbon emissions trading and green financial instruments synergistic? -Comprehensive quantitative research based on content analysis
Source: PLoS One. 2024 Mar 7;19(3):e0298601. doi: 10.1371/journal.pone.0298601 (PMC10919601; doi:10.1371/journal.pone.0298601)
Supplement: S1 Data — (ZIP) [file pone.0298601.s001.zip › Data/地级市Z_F工作报告.zip!/╣π╢1⁄2/╓╨╔╜╩╨/└┤╫╘╣1⁄2╓┌║┼í╛┬φ┐╦╩2╛▌═°í┐_2008.docx]

2007年工作回顾

2007年是本届政府的开局之年。在省委、省政府和市委的正确领导下，在市人大及其常委会和市政协的监督支持下，市政府高举中国特色社会主义伟大旗帜，坚持以邓小平理论、“三个代表”重要思想为指导，以科学发展观统领经济社会发展全局，深入贯彻党的十六大和十七大精神，以建设“两个适宜”城市，构建和谐中山，实现富裕安康为工作目标，奋力开拓，迎难而上，经济社会实现了持续快速健康发展，城市综合竞争力迈上新台阶，各项事业全面进步，社会保持和谐稳定，人民生活水平稳步提高。

经济实现又好又快发展。全市生产总值1210.7亿元，增长14.7%，户籍人口人均生产总值84245元（常住人口人均生产总值48395元）。三次产业比例调整为2.9:61:36.1。工业总产值3585.9亿元，增长16.5%；规模以上工业企业经济效益综合指数144.3%，实现利润总额增长40.9%。外贸出口总值181.5亿美元，增长16.3%。地方财政一般预算收入86.1亿元，增长29.6%。全社会固定资产投资396.3亿元，增长14.3%。单位GDP能耗下降3.9%。全市经济处在较快发展的轨道。

产业结构水平进一步提升。产业结构高级化和适度重型化进程加快，五大支柱产业主导作用明显，占规模以上工业总产值52.9%，高新技术产业产值突破1000亿元。装备制造业成长较快，完成产值753.8亿元，增长13.1%。医药产业稳步发展，实现产值61亿元，增长6.3%。自主创新能力进一步增强，名牌带动战略成效显著，新增中国名牌产品9个，占全省新增总量的1/9；新增中国驰名商标5件，占全省新增总量的1/5强；新增国家质量免检产品5个。产业集群效应不断提升，新增3个国家级产业基地、3个省级专业镇。按照“控制增量、消化存量”的原则，实施产业消长平衡策略，产业转移取得实效，己建和拟建11个产业转移工业园。东部开发加快推进，园区建设日臻完善。第三产业保持快速增长势头，实现增加值436.8亿元，增长17.7%，现代物流等生产服务业加快发展。资本市场发展取得新突破，企业上市工作取得成效，培育后备上市企业58家，完成股份制改革6家，申报上市2家，公用集团整体吸收合并上市进入受理阶段。兴业银行、邮政储蓄银行、中山银达等一批金融机构相继开业。旅游业总收入86.1亿元，增长14.2%。房地产稳步发展，新增商品房登记面积657.4万平方米。社会主义新农村建设取得新成效，实现农业总产值56.2亿元，增长3.5%，市财政投入1.5亿元推进“农村五项工程”，农业标准化、产业化、现代化水平和综合生产能力不断提高。

城市基础设施更加完善。北外环路、古神公路、城桂路二期基本建成，新岐江公路、新沙水公路、福源路、小榄快线、东部快线、中山港二期、太澳高速、广珠轻轨有序推进。完成省重点工程500千伏珠海电厂至香山线路工程和德隆等7个输变电工程建设，全市供电总量148.4亿千瓦时。公交优先战略加快实施，更新和新增公交车333辆，客运站场布局进一步优化。投入3.2亿元推进城乡水利防灾减灾工程，干堤达标加固89.9公里。旧城改造力度加大，长堤路（凤鸣路）片区完成改造规划，新泰安市场动工建设。严格土地管理，土地储备达1.8万多亩，积极盘活闲置用地，不断提高土地投资强度和效益。形成覆盖镇、村的城市管理工作网络和多部门综合治理的城市管理联动机制，城市管理综合执法水平不断提高。

生态环境建设取得新进展。空气质量优良率、主要河流水质达标率均达到100%，新增2个全国环境优美乡镇。投入约23亿元建设18家镇区污水处理厂，中嘉污水处理厂二期工程投入使用。投入2000万元推进污染源在线监测系统建设，污染源普查工作全面启动。投入5.9亿元建设北部组团垃圾处理基地。市镇共投入3.1亿元整治内河涌。开展重点行业环境隐患排查，实施主要污染物排放总量控制，二氧化硫和化学需氧量排放量分别削减0.2万吨和0.6万吨。“绿化中山大地”工程成效显著，全市林木覆盖率达25.2%，中心城区绿化覆盖率达39.2%、人均公共绿地达10.9平方米。

内外两个市场拓展取得新成效。利用外资结构不断优化，批准外商直接投资项目271宗，实际利用外资7.9亿美元，其中投资超千万美元项目12宗，新增世界500强投资项目4宗。“零用地招商”成效显著，增资项目370宗，增资额达9.6亿美元。坚持扩大外贸出口与进口相结合，实现外贸进出口总额255.1亿美元。一般贸易出口增速高于全市外贸出口6.7个百分点，机电产品和高技术产品出口分别增长10.1%和8.3%。进出口公平贸易工作机制进一步完善，反倾销预警工作得到加强。保税物流中心建设取得新进展。民营经济迅速发展，共有民营企业2万家，个体工商户13.5万户，规模以上民营工业总产值1127.3亿元，占全市规模以上工业总产值34.8%。

体制改革不断深化。健全公有资产监管制度，积极推进企业改革、重组，公有资产营运体系进一步完善。坚持利益向下，安排1.4亿元对镇区实行“三奖一补”转移支付。积极增加财政收入，实现市级非税收入40.1亿元，增长20%。进一步扩大公共财政覆盖面，基本公共服务、公益事业支出占财政一般预算支出53%。推进投融资体制改革，实行代建制、产权融资等投融资建设模式，成立市交通集团，通过银团贷款方式融资，有效解决重点工程建设资金问题。深化农村综合体制改革，共有67个村实施村级统一核算。规范市场经济秩序，完善产品质量和食品药品安全管理体系。

社会各项事业取得新发展。安排免费义务教育经费1.19亿元，实现本市户籍21万学生义务教育阶段全面免收学杂费和课本费。教育现代化步伐加快，“振兴初中计划”、“学校优质化工程”深入推进。高中阶段毛入学率达89.4%，高等教育毛入学率达46.8%，24个镇区全部成为广东省“教育强镇”。中山纪念中学扩建、市一中初中部迁建工程全面推进。职业教育超常规发展，高等职业技术学院三期、广东理工职业学院工程全面启动。科技综合实力进一步增强，我市第三次获得“全国科技进步考核先进市”称号，成为“国家火炬计划特色产业集群创新（试点）基地”、国家知识产权试点城市、广东省教育部产学研结合示范基地。国家、省、市级工程中心达到137家，专利申请量增长50.9%，11项科研成果荣获省科技、专利奖。文化事业繁荣发展，公共文化设施日益完善，24个镇区文化站全部达到省特级站标准。全市文化作品获得国家级奖18个、省级奖75个。文化产业加快发展，市博览中心项目建设加快，数字电视工程全面启动。创新人才引进、考核、激励政策，拥有3个博士后工作站。公共医疗卫生体系进一步完善，市疾控中心投入使用，第二人民医院、新中医院、人民医院杨郭恩慈大楼、博爱医院三期工程加快建设。新建改建农村（社区）卫生服务站85间，完成年度目标109%，新增1个国家卫生镇，总数达9个。人口计划生育工作呈现良好态势，人口自然增长率为4.37‰。体育竞技水平进一步提高，全民健身运动蓬勃发展。

人民生活素质和水平稳步提高。十项重要民生工程顺利推进。城乡就业创业工作成效显著，新增就业岗位6.1万个，城镇登记失业率2%。积极推进“充分就业社区”创建工作，设立社区（大嫂）工作坊74个，特困家庭子女和退役士兵免费入读市高级技工学校。全市参加城镇社会保险达420万人次，参加农村基本养老保险的农民占应参保人数的66.8%，参加城乡居民住院基本医疗保险人数达52.5万，占应参保人数的55.8%。全面实现“村村通自来水”、“村村通宽带”，完成抗咸二期工程，完成中心城区预制构件公房改造项目25个。大平安格局初步建立，1701个社会治安视频监控点投入使用，村村（社区）建警务室并配备社区民警，全市刑事案件立案数同比下降5%，破案率同比上升6.7%，各类事故宗数、死亡人数分别下降2.4%和10.7%。流动人口和出租屋管理服务工作取得新成效。坚持信访关口前移，把矛盾化解在基层和萌芽状态，社会保持和谐稳定。获得“全国十大最具幸福感城市”、“中国和谐之城”等荣誉称号。采取积极手段稳定物价，居民消费价格总指数上涨控制在4.4%左右。城乡居民收入持续增加，城镇居民人均可支配收入20317元，增长7.5%；农民人均年纯收入10001元，增长11%。

政府自身建设进一步加强。坚持依法治市，落实责任机制和工作机制。主动接受人大法律监督和政协民主监督，认真办理人大议案、建议和政协提案，共办理人大代表议案、建议131件、政协委员提案205件，答复率100%。深化行政审批制度改革，大力推行网上审批，全面启动“行政效率提速”工程。继续推进政务公开，政府门户网站、《政报》、12319行政服务咨询热线、“城市论坛”、市民邮箱等平台不断完善。开通政务投诉统一平台，民意表达渠道不断拓宽。坚持市长接待日等接访制度，受理群众来信来访事项3877宗，办结率达90%以上。加强教育培训，公务员队伍整体素质不断提高。行政效能监察成效显著，廉政建设和反腐败不断深入，政风行风建设进一步加强。

外事、侨务、台务、审计、统计、老干、气象、国防建设、档案、地方志、妇女儿童、残疾人、民族宗教、新闻出版、人防、扶贫、老区建设等各项工作取得了新成绩。

各位代表，过去一年是我市在主动适应国家进一步加强宏观调控、城市竞争日趋激烈的环境下，克服种种矛盾和困难，全面落实科学发展观，坚持继承与创新，不断取得新的发展成就的一年。一年来，我们深刻地体会到：必须始终坚持落实科学发展观，坚定不移地把发展作为第一要务，推动产业结构的优化升级，实现经济又好又快发展；必须始终坚持解放思想，以改革为动力，着力推动体制机制和观念创新，不断增强发展后劲；必须始终坚持统筹协调，推动经济社会全面可持续发展；必须始终坚持彰显“中山特色”，强化自身优势，一切从中山实际出发，既要立足当前，更要着眼长远；必须始终坚持改善民生，让广大人民群众共享改革发展成果。

各位代表，过去一年的成绩来之不易，是省委、省政府和市委正确领导的结果，是全市人民共同奋斗的结果，也是各位人大代表、政协委员监督支持的结果。我谨代表市人民政府向全市广大工人、农民、知识分子、干部职工、驻我市人民解放军指战员、武警官兵、人民警察，向长期关心支持参与中山建设的港澳台同胞、海外侨胞和海内外各界人士表示崇高的敬意和衷心的感谢！

在看到成绩的同时，我们也应清醒地认识到，我市经济社会发展还存在一些突出问题。主要是：经济发展方式亟待转变，产业结构不够合理，自主创新能力不强；城市承载能力和综合服务功能有待加强；城乡和区域经济社会发展不平衡，统筹发展水平有待提升；资源和环境面临较大压力；社会保障水平有待提高；社会管理难度增大，深层次矛盾开始显现。这些问题都需要我们在今后的工作中切实加以解决。

2008年工作安排

2008年是全面贯彻落实党的十七大精神，推动经济社会全面转入科学发展轨道的重要一年。经过改革开放30年来的积累和发展，我市形成了以专业镇为特色、外向型特征明显、高新技术产业和服务业迅速成长的经济格局，发展动力和后劲不断增强，进入转型发展的关键阶段，具备了加快发展、率先发展和协调发展的良好基础。当前，我们面临的发展机遇前所未有，面对的挑战也前所未有，总体上机遇大于挑战。经济全球化趋势继续扩大，国际产业转移深入推进；党的十七大为经济社会发展指明了前进方向，国内经济将继续保持平稳较快发展。同时，世界经济增长的不确定因素增加，贸易摩擦日益增多，国家采取宏观调控政策防止经济偏快转向过热，防止结构性物价上涨演变为明显通货膨胀，我市原有的比较优势逐渐弱化，面临经营成本全面上升、资源环境约束、外贸政策调整、区域竞争加剧等多重压力，前有标兵快速发展，后有追兵加快追赶。对此，我们必须深刻分析新形势新任务，勇于破解发展中的新难题新瓶颈，把科学发展这一当今时代强音，作为一种精神内核贯穿到我市改革开放和现代化建设的全过程，以新一轮思想大解放推动新一轮大发展。要摒弃不思进取、安于现状的思想，始终保持与时俱进、敢为人先的精神；要摒弃片面狭隘、封闭僵化的意识，始终保持放眼全球、海纳百川的眼光；要摒弃畏惧困难、回避矛盾的心理，始终保持知难而进、敢于创新的气魄；要摒弃以物为本、见物不见人的思维，始终保持以人为本、执政为民的宗旨，用发展的眼光、创新的思路和统筹的办法，努力开创全市科学发展、和谐发展新局面。

2008年政府工作的总体要求是：高举建设中国特色社会主义伟大旗帜，深入贯彻落实科学发展观，全面贯彻党的十七大、省委十届二次全会和市委十二届二次全会精神，以建设“两个适宜”城市，构建和谐中山，实现富裕安康为工作目标，坚持解放思想，深化各项改革；坚持又好又快，促进经济发展方式转变；坚持生态优先，推进全面协调可持续发展；坚持统筹兼顾，加快推进城乡一体化进程；坚持以人为本，建设和谐社会。

2008年经济社会发展的主要预期目标是：生产总值增长11%，单位GDP能耗下降3.9%，二氧化硫和化学需氧量分别下降3.1%和18.2%，社会消费品零售总额增长16%，全社会固定资产投资增长10%，外贸出口增长10%，财政一般预算收入增长13%，城镇居民人均可支配收入增长5%，农民人均年纯收入增长10%，居民消费价格总指数涨幅控制在4%左右，城镇登记失业率控制在3%以内，人口自然增长率控制在6.85‰以下。

2008年政府工作的重点是“抓发展，促改革，惠民生，保平安”。“抓发展”就是要实现经济又好又快发展，做到“好字当头、稳中求进、人均领先”。“促改革”就是要狠抓投融资体制和行政体制等方面改革，为发展提供动力。“惠民生”就是要节能减排，创建全国生态市，统筹城乡协调发展，继续实施“十项民生工程”，努力使全市人民学有所教、劳有所得、病有所医、老有所养、住有所居。“保平安”就是要狠抓社会管理，全面建设平安中山。为此，必须着力做好十方面工作：

一、建立现代产业体系，转变经济发展方式

推进产业结构优化升级，促进三次产业协调发展。坚持走新型工业化道路，大力推进信息化与工业化相融合、服务业与制造业相配套，推动高新技术产业在关键技术上、重化工业在规模上、传统产业在品牌上、服务业在生产服务业发展上、农业在综合生产能力上取得新突破，努力培育一批销售额超百亿元企业。

大力发展先进制造业，培育一批新的优势产业。推进先进装备制造、电子信息、健康医药、新材料、光机电一体化、电光源、数字家电、重化工中下游等战略产业和技术发展，2008年实现工业增加值增长12%。重点培育平板显示、汽车电子产品等新兴高技术产业，力争2008年高新技术产品产值增长18%以上。积极运用高新技术和先进适用技术改造提升传统产业，淘汰一批落后产能，推动传统产业从数量规模型向品牌效益型转变。加快建设华南现代中医药城和中药现代化工程技术研究院，充分发挥国家健康科技产业基地的优势，力争在生物医药、医疗器械制造等方面取得新进展。努力将装备制造工业研究院建设成为省内一流的装备制造技术研发中心和成果转化中心，在风力发电、机电控制等关键和共性技术领域取得新突破。

应对要素制约瓶颈，增强经济发展动力。有序推进产业转移，加快完善配套政策，实现区域间产业梯度合作，继续建设好产业转移工业园。加大土地储备和闲置土地处置力度，加强土地预控和经营管理，形成盘活存量、严控增量、管住总量、集约高效的机制。实施城市土地资源的统一规划、开发和管理，保障重大产业项目、重点基建项目和公共服务设施的用地需求，鼓励产业向园区集中，居住向社区集中。全面推行工业用地招拍挂工作，扩大市场配置土地资源的份额。加强电力需求侧管理，确保电力供需平衡。加快中山嘉明电厂扩建、国电中山燃气发电厂、500千伏桂山输变电工程、横门电厂接入系统以及黄圃、三角等热电联产项目建设，完成中山站改造、宝山和菊城等220千伏输变电工程。完善成品油市场应急预警机制，确保成品油稳定供应。

二、增强自主创新能力，做大做强产业集群

完善技术创新支持体系，提升企业核心竞争力。市财政安排2.4亿元作为工业、科技、服务业、农业企业发展的专项扶持资金，充分发挥政府资金的导向、拉动和放大效应，推动产业向高集聚、高科技、高附加值转变。大力增加固定资产投资尤其是企业技改投资，支持一批重点技术改造项目，鼓励企业通过引进先进设备和技术，以技术结构升级带动产业结构升级。实施“科技强企支撑计划”，引导和支持企业建立工程（技术）中心，大力发展产学研技术产业联盟。积极开展知识产权保护工作，鼓励企业和行业协会参与制定行业标准、国家标准和国际标准，培育检验检测平台。深入推进品牌战略，推动现有品牌形成更强的技术创新支撑能力，扶持做大一批品牌企业，探索建立中山品牌研究院。

推动专业镇整合创新，提升产业集群水平。要把专业镇的创新能力建设作为应对经营成本上升的重要举措，进一步完善产业链、服务链和政策链，实现专业镇向现代产业集群发展。发挥国家级产业基地的集聚作用，鼓励专业市场建设。完善促进产业集群发展的配套服务体系，着力构建面向产业集群内部广大中小企业的技术研发应用、信息服务、人员培训等公共服务平台，推动中小企业联手打造区域品牌。加强专业镇公用基础设施及需求的研究和投入，整合现有创新资源，探索在组团建立共性技术创新平台，加强镇区产业衔接，逐步形成灯饰、家电等特色产业带。

三、加快现代服务业发展，培育新的经济增长点

加快建设物流聚集区，构建购销专业化流通体系。积极推动四大组团物流集聚区的基础设施建设，形成珠江口西岸的区域性物流中心。通过分离、整合、发展等方式，大力培育专业销售企业和专业采购组织等流通主体，重点规划、建设好专业市场、专业购销网络等流通平台，进一步完善资金、人才和政策等支撑，加快构建工业购销专业化流通体系。

建设中心商务区，积极发展“总部经济”。充分利用“孙中山故乡”这一品牌以及良好的人居环境、完善的产业配套，出台相关政策，吸引名优企业将总部设在中山，发展“总部经济”。推进会展业的整合创新，提升会展品牌效应和竞争力。加快火炬区、东区、小榄等中心商务区建设，吸引风险投资、证券交易、法律服务、信息咨询、创意策划等服务企业进驻，发展“都市经济”。完成长堤路（凤鸣路）商圈改造，推进富华、怡华、大信等特色商圈和轻轨站场商务区建设，进一步优化商业网点布局，打造商贸服务业集聚发展平台。

加快金融业发展，打造资本市场新平台。推动企业发展由间接融资为主向间接融资与直接融资并重转变，大力培育企业上市后备资源，促进一批科技含量高、增长速度快、盈利能力强的企业，尤其是品牌工业制造企业上市发展，通过资本市场培育龙头企业。进一步优化金融生态环境，吸引股份制商业银行和外资银行到中山发展，为我市提供更完善的资本平台、更丰富的金融产品，鼓励外资和民间资本参与城市基础设施建设和社会事业发展。加快发展股权投资和担保业务，强化防范金融风险预警机制。加快产权交易市场建设。大力发展保险业。

促进旅游业发展，扩大消费需求。以旅游业为龙头，全面推进生活服务业发展。通过引入国际资本，推动旅游资产重组和资源整合，深化产权制度、经营机制和管理体制改革，实现旅游业从景点经营向线路经营转变，从景点开发向板块开发转变，从产业经营向产业经营与资本运营相结合转变，从观光旅游为主向文化、产业、观光、休闲度假相结合的综合旅游转变。全力打造新的旅游板块，积极创建翠亨和北台两个5A级国家旅游景区。增强居民消费能力，大力培育信息、汽车、文化、健康等消费热点，加快推进消费结构升级。加强消费者权益保护，实施好“菜篮子”工程，加强重要生活商品的生产、供应和市场调控，保持价格水平基本稳定。

四、加强区域合作，完善开放型经济体系

加快转变外贸增长方式，壮大民营经济实力。创新利用外资方式，优化外资结构。坚持大项目、大产业带动战略，强化项目跟踪服务，着重引进世界500强企业和国内大型企业。推广园区招商、产业链招商、规划招商和股权招商。研究制定外贸奖励政策，优化对外贸易结构，进一步提高出口商品附加值，大力发展一般贸易，促进进出口贸易协调发展。大力发展保税物流业务，推动加工贸易从传统制造业为主向中高端制造业为主转变，从国外市场到国内外市场并重转变。注重引进、消化、吸收和创新，增强加工贸易的辐射、带动能力。引导企业积极应对反倾销和贸易壁垒，积极开拓东盟、中东、南美等新兴市场。支持民营资本进入公用事业、基础设施和金融服务等行业。构建面向中小企业的技术创新平台和社会化服务体系，鼓励民营企业创立品牌、创新技术、改革体制、规范管理。拓宽中小企业融资渠道，加快完善信用担保和再担保体系，支持有条件的中小企业上市直接融资。大力发展创业投资。实施“中小企业成长工程”，为民营企业做大做强提供良好环境。

探索高效的区域合作体制，加快组团发展。突出市级对规划资源的集中统筹，主动应对和研究主体功能区划分问题，加强各类规划与四大组团功能的衔接和协调，建立主题明确、脉络分明、重点突出、体制科学的城镇规划体系。按照区域一体化发展的思路，强化资源整合与部门协调，共同推动区域经济协调发展，突出东部组团的带动示范作用。健全组团发展协调机制，建立西北组团联席会议制度，加大对跨组团基础设施建设的统筹力度，加快构筑组团之间的快速交通网络，实现组团之间基础设施的“无缝”对接。

加强产业对接，完善区域产业配套。按照“共享、共赢、错位、互补”的原则，实施“外联内合”战略，积极开展区域经济合作。坚定不移地推进东部开发，抓住产业结构调整这个核心，建设产业发展新高地。加快临海工业园和东部科技产业带建设，努力打造高层次的高新技术产业集聚区，积极吸引高端服务业。加强与周边地区石化工业的衔接，大力发展污染小、占地少、附加值高的化工中下游产业。南部要积极发展出口加工业。西北部要加强与周边城市产业和基础设施的协调，通过实行产业差异化战略，实现家电等产业的整合与对接。

五、积极建设生态文明，力争实现全国生态市创建目标

推进节能减排，大力发展循环经济。牢固树立生态文明观念，把建设资源节约型、环境友好型社会放在工业化、城市化发展的突出位置。严格落实节能减排目标责任制，加强对产业和投资项目的能耗监测和统计，探索差别化价格政策，支持企业开展节能、节水、节材和资源综合利用等方面的技术改造，努力形成节约型产业结构、增长方式和消费模式。积极发展环保产业，推进循环经济工业园区试点建设。试点推广一批可再生能源利用项目，加快转移和淘汰一批环境承载压力大的劳动密集型、环境污染型企业。全面推行清洁生产，引进优质清洁能源，推进市域天然气利用及火力电厂“油改气工程”与“脱硫工程”，新增省清洁生产示范企业10家。

增强城市可持续发展能力，从源头上防治污染。继续深入推进岐江河综合整治工程，市财政投入1亿元继续开展内河涌整治，着力抓好内河支堤加高培厚与河道清淤疏浚工程。严格实行主要污染物排放总量控制，严厉整治违法排污企业。健全污染源监督长效机制，全面实施重点污染源在线监控，加强汽车尾气、河流水质和空气质量监控。

加快污水处理厂建设，全面落实生态市创建目标。加大生态建设投入，建成镇区污水处理厂18家，新增污水日处理能力46.5万吨，城区生活污水集中处理率达80%以上、镇区达70%以上。完成中嘉污水处理厂二期、珍家山污水处理厂首期污水管网收集系统建设，提高污水截流率和处理率。建设北部、南部组团垃圾处理基地和全市固体废物综合处理中心，生活垃圾无害化处理率达100%。继续积极推进全国环境优美乡镇创建工作。实施五桂山生态保护规划，推进林相改造第四期工程，加大湿地保护和红树林种植力度，规划建设古香林公园、名树园等。推进生态文化建设，提高市民的生态意识和环保意识。

六、统筹城乡发展，深入推进社会主义新农村建设

深化农村综合改革，落实强农惠农政策。全面推进城乡统筹协调发展，按照“多予、少取、放活”和“工业反哺农业、城市支持农村”的方针，进一步建立以工促农、以城带乡的长效机制，加大对农业农村发展的投入力度。完善农村股份合作制改革，加快推进村级统一核算。把握机遇，完善新一轮农村土地承包管理体制，积极推进土地承包经营权依法有序流转。探索新的农村建设投融资体制，改善农村金融服务，探索推进农业保险。

以促进农民增收为核心，积极探索农村集体经济有效实现形式。引导和支持农村发展壮大各种新型社会化服务组织和专业合作经济组织，构建“共建、共享、共赢”的农村发展利益共同体，加快发展新型村级集体经济。坚持“宜工则工、宜农则农”，进一步扶持欠发达村发展经济，市给予土地指标支持，镇村两级共同投入发展物业经济，市财政连续两年每年给予每个欠发达村50万元集体经济项目补贴资金，增强“造血”功能，确保2009年中期所有欠发达村集体经济收入达到100万元以上。加强涉农收费项目监管，继续做好农民减负工作。

转变农业发展方式，大力发展现代农业。优化农产品区域布局，加快农业科技创新和推广，推进农业、农村信息化建设。推进农业产业化经营，发展农产品精深加工，加强农产品市场建设，扶持农产品物流业发展，培育壮大农业龙头企业，办好首届中国（中山）南方绿化苗木博览会。安排1300万元健全农业标准体系、检测体系和质量认证体系，强化农产品质量安全监管和动植物疫情防控，积极发展效益农业、特色农业、品牌农业和绿色农业。建设一批现代农业示范基地，推动现代高效农业规模化发展。加强农村基础设施建设，年内完成2.7万亩连片耕地的农路农桥建设，市财政安排5000万元完成250公里主干农路的硬底化建设。加强基本农田保护，确保粮食生产稳定。加强海洋资源管理。

完善农村公共设施和公共服务，全面推进城镇化进程。积极推进中心镇、村的规划建设，今明两年完成村级规划编制。稳步推进“村改居”工作和城中村改造，大力发展村民公寓社区，引导农村居民转变居住和生活方式。健全农村社会保障体系，有效解决农民后顾之忧。创新农民培训方式，全面提高农民素质，培育有文化、懂技术、会经营的新型农民，发挥农民建设社会主义新农村的主体作用。

加强农村基层建设，健全村民自治制度。规范农村集体经济组织管理和资产、财务管理，加强农村审计监督，推动村级社会管理事务与经济事务分离，完善农村基层干部激励保障机制。加强对农村选举工作的指导，依法做好村级换届选举工作。

七、加快城市建设，进一步完善城市功能

推进城市基础设施建设，提高城市承载和服务水平。按照“内通外拓”的思路，积极构建干线公路网，努力实现与周边城市道路的“无缝”对接，加快建设“半小时生活圈”。推进新沙水公路、新岐江公路、福源路建设及中拱公路改造，开展小榄快线、中番大桥、神湾二桥的前期工作。配合广珠城际轨道交通建设，制定轻轨站场规划和市内轻轨交通规划。整治洪奇沥、磨刀门水道，完善内河航道运输网。积极实施“公交优先”发展战略，完善财政补贴公交发展制度，年内更新和投放公交车300辆以上，新建黄圃、城北和中山港三个汽车客运站，加快城区四大公共停车场建设。年内建成220个农村候车亭，争取在2009年底前实现“镇镇有站、村村有亭”的目标。加强旧城改造，完成岐江桥重建，加快人民医院片区、怡华商圈道路改造，规划建设博爱路银通街及悦来南路下穿车行隧道。城乡水利防灾减灾工程要实现重大突破，扩大长江水库库容，推进中顺大围达标加固、西河水闸重建等项目的主体工程建设，加固干堤30公里。完善城市公共设施体系，确保市博览中心综合展馆投入使用，加快中心粮库二期工程建设，规划建设粮食物流中心。完成博爱医院三期、第二人民医院工程，推进新社会福利院、新中医院、人民医院杨郭恩慈大楼建设和泰安、东门、南门市场改造。投入5亿元加快东部组团基础设施建设，加快东部快速路网、黄圃港区一期工程、保税物流中心和石油化工储备区建设。启动中山港二期两个5000吨级多用码头的前期工作。

完善政府项目投融资管理，加快重点项目建设步伐。按照“储备一批、准备一批、立项一批”的编制思路，创新项目编制管理办法，科学编制年度投资计划，优化重点项目审批流程，加强对履行政府职能的中介机构的管理，加快重点项目协同办公系统建设，实现重点项目提速。创新投融资体制改革，按照“统筹分类、试点先行、模式推广”的思路，重点推行BT、BOT、项目代建制、银团贷款、收费质押等新型项目融资和组织方式，启动市场化代建试点工作，逐步建立“政府主导、社会参与、市场运作”的投融资新格局。加大征地拆迁力度，研究制定拆迁补偿新标准及相关政策，确保重点项目按计划开工建设和竣工投产。

八、深化财税管理体制改革，完善公共财政体系

加强税收征管，加大公共财政对社会建设的投入。依法开展税收征管工作，积极培育税源和新的税收增长点，规范非税收入收支管理。调整优化财政支出结构，把57%的财力用于公共服务和公共产品，将新增财力重点投向公共社会事业和新农村建设等领域，其中20%用于增加社会保障事业的投入。坚持过“紧日子”，积极推行“以钱养事，以事定费”的公共服务采购政策。加强预算监督和市镇两级财政性债务管理，形成财政风险控制机制。完善财政支出管理，深入推行财政支出绩效评价。

健全市镇两级财政管理体制，完善分类转移支付。新增横栏、三角、黄圃三个镇实行限期2年的一级财政，对民众、阜沙、神湾、大涌四个镇加大转移支付力度，增强欠发达镇区自我发展能力。提高镇区依法理财水平，编制全面、完整、细化的综合预算，规范土地出让收支。加强市级财政资金清收管理，做好重点项目资金保障工作。

深化公有资产管理体制改革，确保公有资产保值增值。坚持“一改二清三重组”思路，推动市属企业改革和发展。以市场化为目标，减少政府对经济领域的直接参与，努力构建“小政府、大社会”。加强市属国有资产监管，重要公共资源经营统一纳入政府资源管理。推进公有物业管理体制改革，整合公房资源，打造城市建设投融资新平台。

九、推进“文化名城”各项建设，提升城市文化竞争实力

坚持教育优先，建设人力资源强市。加快推进教育现代化进程，继续实施“振兴初中行动计划”、“学校优质化工程”和“名校带动战略”，推进义务教育均衡化、优质化发展。通过“扩容促优”普及高中阶段教育，推进示范性高中建设，完成纪念中学和实验高中扩建、一中初中部迁建。规范学校办学行为，支持民办学校健康发展。提高教师待遇，强化教师队伍建设。完成广东理工职业学院、西北组团职业学校、中专迁建、中山职业技术学院三期，启动市职业教育实训基地建设工程。促进高等教育发展，支持电子科大中山学院和广东药学院中山校区做大做强。继续实施“人才强市”战略，落实人才政策，创新人才管理、奖励、培养机制，逐步形成引进、留住和用好人才的良好环境。

增强文化发展活力，促进文化繁荣。全面推进孙中山文化、历史文化、产业文化、民俗文化、公共文化、博爱文化、“三名”文化、生态文化等“八大文化工程”建设。积极筹建博物馆群、档案馆、新市工人文化宫等公益性重点文化设施，凸显城市形象和文化特色。加强社区和农村文化设施建设，推进图书馆分馆、社区（村级）文化室建设，扩大公共文化服务覆盖面。切实保护文化遗产资源，重点加强孙中山研究和资源利用，做好申报“中国历史文化名城”的各项基础性工作。做大做强文化产业，以创建国家、省文化产业示范基地为突破口，推动国家级游戏游艺产业基地、中国包装印刷基地、小榄镇文化艺术品产业基地、三乡镇古玩产业基地、坦洲镇首饰文化产业基地等特色文化产业基地建设。鼓励文化精品创作，培育文化产业骨干企业和文化名家，打造文化产业品牌。加快推进数字电视整体转换工作。发展产业媒体，丰富产业文化。以迎奥运为契机，促进全民健身和竞技体育运动深入发展。

大力弘扬新时期中山人精神，建设中山人精神家园。坚持正确舆论导向，弘扬社会正气，增强市民爱国主义、集体主义、社会主义信念，建设社会主义核心价值体系。大力倡导基本道德规范，加强社会公德、职业道德和家庭美德教育，进一步加强未成年人思想道德建设，积极开展群众性道德实践活动。巩固全国首批文明城市成果，弘扬和充实“博爱、创新、包容、和谐”的新时期中山人精神。以纪念改革开放30周年和庆祝中山升格为地级市20周年为契机，深入推进各项精神文明创建活动，提高城乡文明程度。

十、加强社会建设，注重改善民生

深化改革，发展社会各项事业。进一步明确社会事业领域中不同主体的职责，积极稳妥、分类推进科技、文化、教育、医疗卫生管理体制改革，加快实现政事分开、管办分离。完善“政府购买服务”的模式，积极建立以政府投入为主导、社会各界共同投入兴办社会事业的新机制和多种筹资模式。加快建设社会信用体系，继续规范和发展各类行业协会、商会及社会中介组织，完善社会中介组织的准入制度。健全市场监管体制，整顿和规范市场经济秩序。

实施积极的就业政策，促进以创业带动就业。把就业与创业结合起来，把工资性收入与财产性收入结合起来，完善创业扶持政策，设立青年创业基金，推动城乡劳动者能创业、创稳业、创好业。市财政安排2000多万元作为就业再就业专项资金，重点帮助困难群体就业，大力开发公益性岗位，推广社区（大嫂）工作坊等就业方式。继续实施城乡青年技能培训、创业富民工程和技能人才振兴计划，培训城乡劳动者5万人次。探索建立市场化培训运作模式，引进品牌培训机构，构建政府购买培训成果平台。

加快建立覆盖城乡的社会保障体系，提高社会保障水平。加快推进金保工程，探索建立社会保障财政转移支付机制，推动欠发达镇区参保，年内安排2.17亿元专项资金用于社会保障工作，力争实现社会保险参保达430万人次以上。加快推进农村基本养老保险，力争年内基本实现农村基本养老保险全覆盖，鼓励具备条件的农村居民参加城镇社保。引导房地产业健康发展，建立多层次住房供应体系，投入2亿元积极推进经济适用房、廉租住房建设，逐步推行以租金补贴为主、实物配租和租金核减为辅的廉租住房保障体系。年内完成第三期低保家庭危房改造和第二期城区预制构件公房改造工程。健全城乡统筹的社会救助体系，安排2180万元用于城乡社会救济和最低生活保障。继续做好双拥、优抚安置工作。倡导社会互助，办好慈善总会，发展残疾人事业，加强老龄工作。

加快建立覆盖城乡的基本医疗卫生制度，提高全民健康水平。强化政府投入，建设覆盖城乡居民的公共卫生服务体系，为群众提供安全、有效、方便、价廉的医疗卫生服务。全面实施城乡居民住院基本医疗保险，完成农村合作医疗向城乡居民住院基本医疗保险并轨，力争年内实现城乡医疗保险全覆盖。启动实施门诊基本医疗保险制度。积极完善农村（社区）公共卫生服务体系建设，不断完善市、镇、村三级公共卫生网络，完成农村（社区）卫生站建设目标。深入开展爱国卫生运动，发展妇幼事业。把产业转移与调整人口素质结构有机结合起来，完善流动人口管理办法，建立控制人口增长的长效机制，建立健全计生层级动态管理责任制和利益导向型人口控制机制，稳定低生育水平，继续推进出生缺陷干预工程。

完善社会管理，维护大平安格局。健全各级社会管理体制，进一步完善适应社会转型的利益协调、诉求表达和权益保障机制，妥善处理人民内部矛盾。构建以人民调解为主线的“大调解”格局，完善信访工作网络，畅通信访渠道，提高处理信访问题和群体性事件的水平。全面落实《劳动合同法》、《劳动争议调解仲裁法》，逐步在镇区一级建立协调劳动关系三方机制，保障劳动者合法权益。加强社会组织建设和管理。抓好普法和法律援助工作。以平安社区建设为重点，深入开展平安创建工作，有效维护公共安全。完善覆盖城乡的社会治安立体防控网络系统。重拳打击各类多发性、严重暴力犯罪，着力整治治安重点地区和突出治安问题。进一步健全监管网络，强化安全生产管理和监督，确保安全生产事故及经济损失继续下降。

建设人民满意的政府

坚持科学行政，加快政府职能转变。正确处理管理与服务的关系，努力形成行为规范、运转协调、公正透明、廉洁高效的行政管理体制。优化公共资源配置，把政府工作、政策支持、财力保障的重点逐步向社会事业、公共事业倾斜，不断提高社会管理、应急保障和公共服务水平。深化审批制度改革，创新行政审批模式，加强电子政务建设。推动行政效率提速，严格办事时限要求，探索精简合并政府职能和政府工作流程的优化再造。加强政府门户网站资源整合及“城市论坛”等平台建设，倾听民生，汲取民智。全面推行目标管理，形成“年初有计划，月月有检查，年终有考核，全年抓落实”的有效工作机制，探索开展政府部门绩效评估试点工作。

坚持依法行政，切实提高政府执行力和公信力。主动接受人大的法律监督和政协民主监督，畅通民意表达渠道。深入贯彻落实行政许可法和依法行政实施纲要，推行行政问责制，探索建立行政过错责任追究制度，强化对行政权力的监督和制约。规范执法行为，提高执法水平，努力建设法治政府。大力实施“阳光工程”，完善重大决策规则和程序以及重大决策配套政策形成机制，完善新闻发言人制度和征询意见、专家论证、市民听证等制度，使机关运作更加公正诚信、透明便民。

坚持学用结合，努力建设学习型政府。加强公务员教育和培训，广泛开展学习活动，丰富充实贯彻科学发展观的知识，不断提高驾驭市场经济、推进自主创新、加强社会管理、促进依法办事的能力，创新发展理念，破解发展难题，以学习的突破促进思想的解放，以思想的解放推动发展的跨越。

坚持从严治政，确保廉洁从政。深入推进反腐倡廉工作，紧密结合行政审批、财税金融、投资体制、国有资产管理、人事制度等各项改革，建立健全从源头上防范腐败的机制。强化行政效能监察、执法检查和审计监督，严肃查处违法违纪案件，坚决纠正损害群众利益的不正之风。继续保持奋发有为的精神状态，大兴敢于坚持原则、敢于严格管理、敢于批评错误、敢于改革创新之风。精简文山会海，规范公务接待，降低行政成本，建设节约型政府。打造一支政治坚定、业务精通、清正廉洁、作风优良的公务员队伍。
